# Effect of Hydrogen Exposure on Mechanical and Tribological Behavior of Cr_x_N Coatings Deposited at Different Pressures on IN718

**DOI:** 10.3390/ma10050563

**Published:** 2017-05-20

**Authors:** Aleksei Obrosov, Alina N. Sutygina, Alex A. Volinsky, Anton Manakhov, Sabine Weiß, Egor B. Kashkarov

**Affiliations:** 1 Chair of Physical Metallurgy and Materials Technology, Brandenburg Technical University, Cottbus 03046, Germany; sabine.weiss@b-tu.de; 2 Department of General Physics, National Research Tomsk Polytechnic University, Tomsk 634050, Russia; sutygina2013@mail.ru (A.N.S.); egor_kashkarov@mail.ru (E.B.K.); 3 Department of Mechanical Engineering, University of South Florida, Tampa, FL 33620, USA; volinsky@usf.edu; 4 Laboratory of Inorganic Nanomaterials, National University of Science and Technology “MISiS”, Moscow 119049, Russia; ant-manahov@ya.ru

**Keywords:** Cr_x_N coatings, PVD, hydrogenation, tribology, mechanical properties, GDOES

## Abstract

In the current study, the properties of the Cr_x_N coatings deposited on the Inconel 718 superalloy using direct current reactive magnetron sputtering are investigated. The influence of working pressure on the microstructure, mechanical, and tribological properties of the Cr_x_N coatings before and after high-temperature hydrogen exposure is studied. The cross-sectional scanning electron micrographs indicate the columnar structure of the coatings, which changes from dense and compact columns to large columns with increasing working pressure. The Cr/N ratio increases from 1.4 to 1.9 with increasing working pressure from 300 to 900 mPa, respectively. X-ray diffraction analysis reveals a change from mixed hcp-Cr_2_N and fcc-CrN structure to approximately stoichiometric Cr_2_N phase. After gas-phase hydrogenation, the coating deposited at 300 mPa exhibits the lowest hydrogen absorption at 600 °C of all investigated coatings. The results indicate that the dense mixed cubic and hexagonal structure is preferential for hydrogen permeation resistance due to the presence of cubic phase with higher packing density in comparison to the hexagonal structure. After hydrogenation, no changes in phase composition were observed; however, a small amount of hydrogen is accumulated in the coatings. An increase of coating hardness and elastic modulus was observed after hydrogen exposure. Tribological tests reveal that hydrogenation leads to a decrease of the friction coefficient up to 20%–30%. The best value of 0.25 was reached for hydrogen exposed Cr_x_N coating deposited at 300 mPa.

## 1. Introduction

Since the commercialization of TiN coatings in 1980s, transition metal nitride hard coatings have been extensively applied in bearings, gears, as well as cutting and forming tools because of their high hardness, good wear, and corrosion resistance [1,2]. Their capability to extend tool lifetime in abrasive and corrosive environments has been verified [3].

Chromium nitride coatings exhibit higher corrosion and oxidation resistance in comparison to other nitride coatings [4,5]. Moreover, Cr–N coatings have attracted much attention in different applications in terms of their high temperature stability, chemical inertness, high toughness, and a friction coefficient lower than TiN [6].

Cr–N coatings can be synthesized by various PVD processes, such as hollow cathode discharge [7], pulsed laser deposition [8,9], ion-beam-assisted deposition [10,11], arc ion plating [12,13,14], and magnetron sputtering [15,16,17,18]. 

Phase structure, morphology, and mechanical properties of Cr–N coatings deposited using magnetron sputtering strongly depend on the deposition parameters, such as working pressure, bias voltage, substrate temperature, target power, substrate frequency, nitrogen flow rate, etc. [4,18,19,20,21]. Among them, working pressure is one of the key parameters which controls the deposition process. Gas pressure directly affects phase structure, preferred orientation, chemical composition, and deposition rate of the coatings [22,23]. 

Application of Cr–N coatings and Inconel 718 (IN718) superalloy in the hydrogen-contained aggressive environments requires detailed knowledge about changes in physical and mechanical properties of the material during hydrogen interaction at elevated temperatures. According to literature, the presence of δ-phase in IN718 at high hydrogen concentrations dramatically reduces the ductility of the alloy [24]. Furthermore, hydrogen embrittlement of IN718 occurs preferably at the grain boundaries even at low hydrogen concentrations. Thus, developing hydrogen-resistant coatings with adequate mechanical and tribological properties on the IN718 alloy is also very important to prevent hydrogenation.

Despite numerous publications about Cr–N films, up to now the effect of hydrogenation on mechanical and tribological properties of Cr–N coatings has not been completely understood. The objective of this paper is to investigate the influence of the pressure on microstructure, mechanical, and tribological properties of Cr_x_N coatings before and after high-temperature hydrogen exposure.

## 2. Experimental

Cr_x_N coatings were fabricated using DC magnetron sputtering in the CC800/9 industrial coater from CemeCon AG (Würselen, Germany). The coatings were deposited onto IN 718 and (100) silicon wafers. IN 718 substrates were mirror polished, ultrasonically cleaned in acetone and ethanol, and then placed in the chamber at the substrate-to-target distance of 70 mm. For deposition of the coating, with the stationary table, the specimens were placed opposite to the target. A high purity, single Cr target (99.99%) from CemeCon AG (Würselen, Germany) was used. Prior to sputtering, a base pressure of less than 8.0 mPa was achieved in the chamber. Then the substrates were etched with Ar^+^ plasma at a bias voltage of −650V for 30 min in order to remove surface contaminations and ensure proper adhesion of the deposited films. The coating temperature was kept constant through all experiments at 500 °C. The target power of 2 kW and substrate bias voltage of 90 V were kept constant. N_2_/Ar ratio for all deposited coatings was 0.23. In this study, the working pressure was changed from 300 to 900 mPa. 

The film thickness was measured using the CemeCon AG (Würselen, Germany) calowear test machine. The deposition rate was calculated from the film thickness and the corresponding deposition time. The cross-section images of the Cr_x_N thin films deposited on Si wafers were analyzed by means of scanning electron microscope from Tescan (Brno, Czech Republic). The chemical composition of the Cr_x_N layers was determined by wavelength-dispersive X-ray spectroscopy (WDS).

The hydrogenation was carried out in gas atmosphere using the Gas Reaction Controller (GRC) technique (Pittsburgh, PA, USA). Initially, the sample was placed into the vacuum chamber and evacuated to the base pressure of 10^−3^ Pa. Then, the sample was heated to 600 °C with a heating rate of 6 °C/min. Finally, the chamber (175 cm^3^) was filled with hydrogen (99.999% purity) up to 2 atm pressure and kept for two hours. After hydrogenation, hydrogen was pumped out of the chamber during slow cooling. Elemental distribution was analyzed by glow-discharge optical emission spectroscopy (GDOES) using GD Profiler 2 (Horiba, Japan).

Crystallographic phases and XRD patterns of the coatings were identified using the Shimadzu XRD 7000S (Kyoto, Japan) equipped with the OneSight wide-range high-speed detector at 40 kV and 30 mA with Cu*K*_α_ radiation (λ = 0.15406 nm). Nanohardness of the coatings was measured using the nanohardness tester NHT-S-AX-000Х from CSEM (Neuchatel, Switzerland). This device analyzes changes in load and indenter penetration depth at the loading–unloading cycle using the Oliver and Pharr method [25]. The load was adjusted so that the penetration depth did not exceed one-tenth of the coating thickness [26]. The average of 20 measurements was calculated. The evolution of the friction coefficients was investigated by the high-temperature tribometer TNT-S-AH0000 from CSEM (Neuchatel, Switzerland) under dry friction conditions with a 5 N vertical load and a linear sliding speed of 2.5 cm/s for 15,000 laps (the total distance is approx. 150 m).

## 3. Results and Discussion

### 3.1. Morphology and Structural Analysis

**Figure 1:**
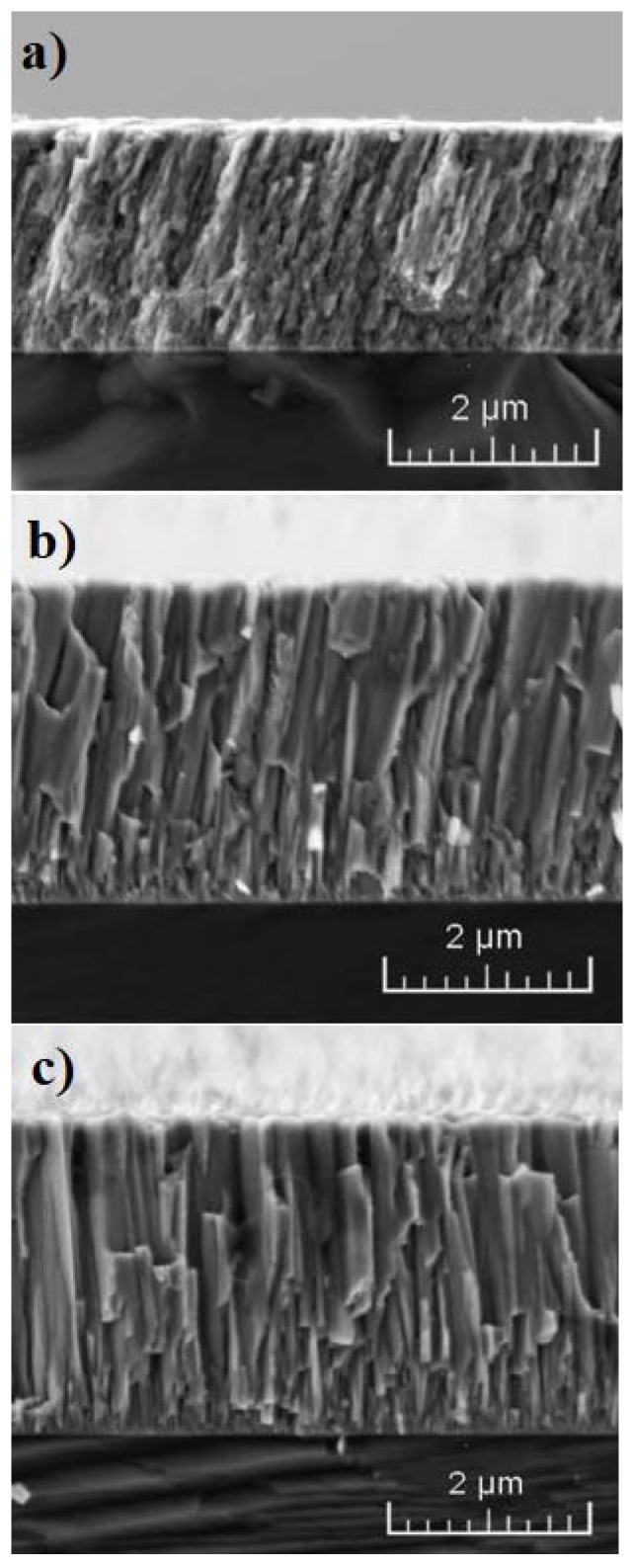
Cross-section SEM images of Cr_x_N deposited at various pressures (**a**) 300 mPa; (**b**) 600 mPa; and (**c**) 900 mPa.

The cross-sectional SEM micrographs of the Cr_x_N coatings, deposited at various pressures are shown in Figure 1. It can be observed that the coating deposited at a low pressure (300 mPa) has a compact columnar microstructure with closely-packed columns. The increasing working pressure results in larger columns and higher porosity of the coating. Furthermore, a change in coating growth was observed. At a low pressure, the coating grows in approximately 65° direction with respect to the substrate, whereas the coating growth at 900 mPa was practically perpendicular to the substrate. Coating microstructure plays an important role in affecting mechanical and tribological properties as well as hydrogen permeation [18,27,28,29,30]. 

The WDS analysis shows that the Cr content increases from 59 at. % to 66 at. % with increasing pressure, while the nitrogen content decreases (Table 1). The Cr/N ratio changes from 1.4 to 1.9. At these compositions, according to the equilibrium phase diagram [31], a change from mixed CrN + Cr_2_N to uniform Cr_2_N can be expected. 

An increasing coating deposition rate was found for Cr_x_N coatings with increasing chamber pressure from 70 nm∙min^−1^ at 300 mPa to 115 nm∙min^−1^ at 900 mPa. On the one hand, these results correlate with the trend that at low pressures there is less ionization and, therefore, a lower deposition rate is observed [32]. On the other hand, the deposition rate could further decline with pressure as a result of resputtering and decrease of mean free path in the deposition chamber [33,34]. Benhenda et al. [35] found that the deposition rate first increases and then decreases with increasing pressure. Furthermore, phase composition could also be affected by pressure. Hones et al. [36] indicated that the Cr_2_N phase has a higher growth rate than the CrN phase.

### 3.2. Gas-Phase Hydrogenation

**Figure 2:**
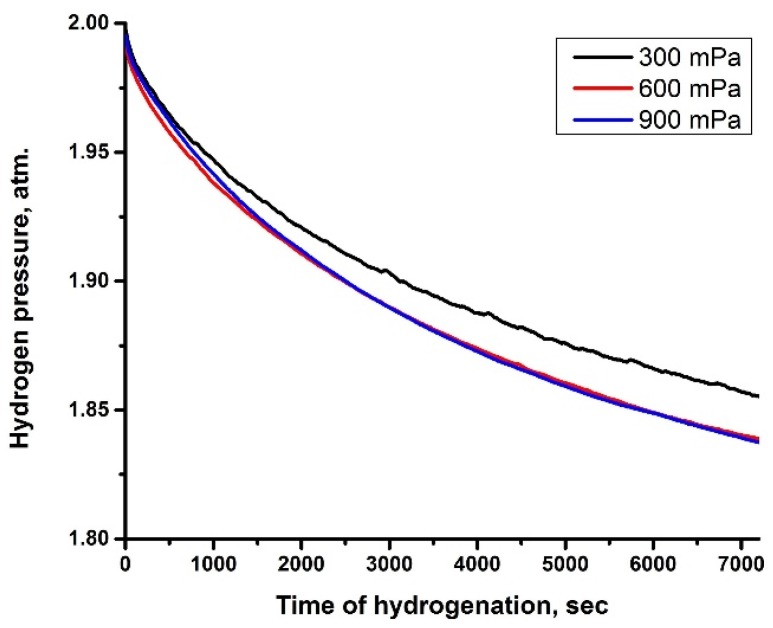
Hydrogen pick-up curves of IN718 with Cr_x_N coatings for hydrogenation at 600 °C.

Gas-phase hydrogenation was carried out to investigate hydrogen resistance properties of the Cr_x_N coatings deposited on the IN718 alloy, along with changing mechanical and tribological properties after high-temperature hydrogen exposure. The decrease in hydrogen pressure in the chamber indicates that the absorption process is taking place. The slope of the curves shows the hydrogen absorption rates of the samples in Figure 2. The absorption curves are not linear: hydrogen absorption rate gradually decreases with hydrogenation time and tends to saturate when the concentration of absorbed hydrogen is maximal for given temperature and pressure conditions.

The lowest hydrogen absorption was observed for the Cr_x_N coating deposited at 300 mPa, which exhibits dense columnar structure. The absorption rate strongly depends on the kinetics of hydrogen adsorption on the surface and hydrogen permeation through the coating. These data indicate that the dense structure of the coatings is preferential for hydrogen permeation resistant coatings. Furthermore, the difference in hydrogen absorption could be associated with the changing crystalline structures of the coatings.

### 3.3. X-ray Diffraction

**Figure 3:**
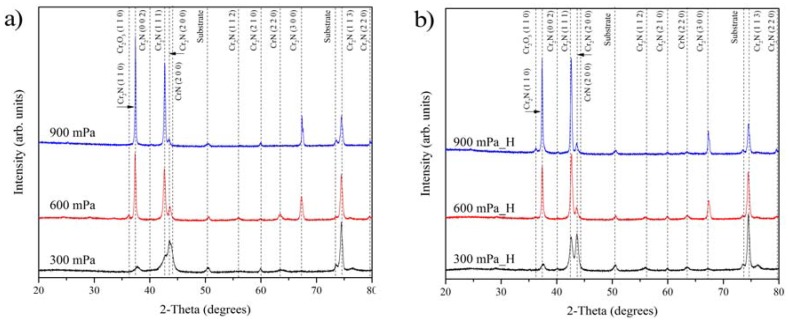
XRD of Cr_x_N coatings deposited at different pressures (**a**) before; and (**b**) after hydrogenation.

In the Cr–N system, three solid phases exist: the solid interstitial solution body centered cubic Cr(N), hexagonal Cr_2_N, and face centered cubic CrN [31,37]. Wei et al. [37] reported that body centered cubic Cr(N) phase could be formed exclusively at a low nitrogen partial pressure (less than 8%), corresponding to coatings with N concentration ≤ 25 at. %. Figure 3a,b show the phase evolution in the Cr_x_N films deposited at different pressures obtained by means of X-ray diffraction before and after hydrogenation. With increasing working pressure an evident trend of phases changing from mixed hexagonal Cr_2_N and face centered cubic CrN structure to approximately stoichiometric Cr_2_N is visible. Three dominant reflections, Cr_2_N (113), (111), and CrN (200) occur in the coating deposited at 300 mPa. The reflections of CrN (200) and Cr_2_N (200) decline at higher working pressure, while Cr_2_N (110) and (111) become dominant reflections (Figure 3a). The increase in the intensity of Cr_2_N (110) and (111) reflections with higher working pressure is due to the increase in deposition rate favoring preferential growth along the (111) orientation [18,23]. The appearance of Cr_2_N (113) and (300) reflections is related to the enhanced mobility of adatoms on the film surface at high deposition temperatures [23].

After hydrogenation, the phase composition remains stable and no new phase occurs. XRD results show that the coating deposited at 300 mPa with the lowest hydrogen absorption has a mixed hcp-Cr_2_N and c-CrN structure. Thus, the cubic CrN phase, formed at lower working pressure with its denser structure could improve the barrier properties against hydrogen permeation. Similar results were reported by Tamura et al. for the BN coatings, where coatings containing mixed cubic and hexagonal BN phases were effective in reducing the rate of hydrogen permeation [29]. It could be concluded that the presence of the c-CrN structure is preferable for hydrogen permeation resistant coatings due to a higher packing density of the cubic structure in comparison to the hexagonal structure [38]. Furthermore, preferred orientation of the deposited films strongly depends on the working pressure and could influence mechanical properties and hydrogen permeation, also [39,40].

### 3.4. Depth Distribution of Elements

**Figure 4:**
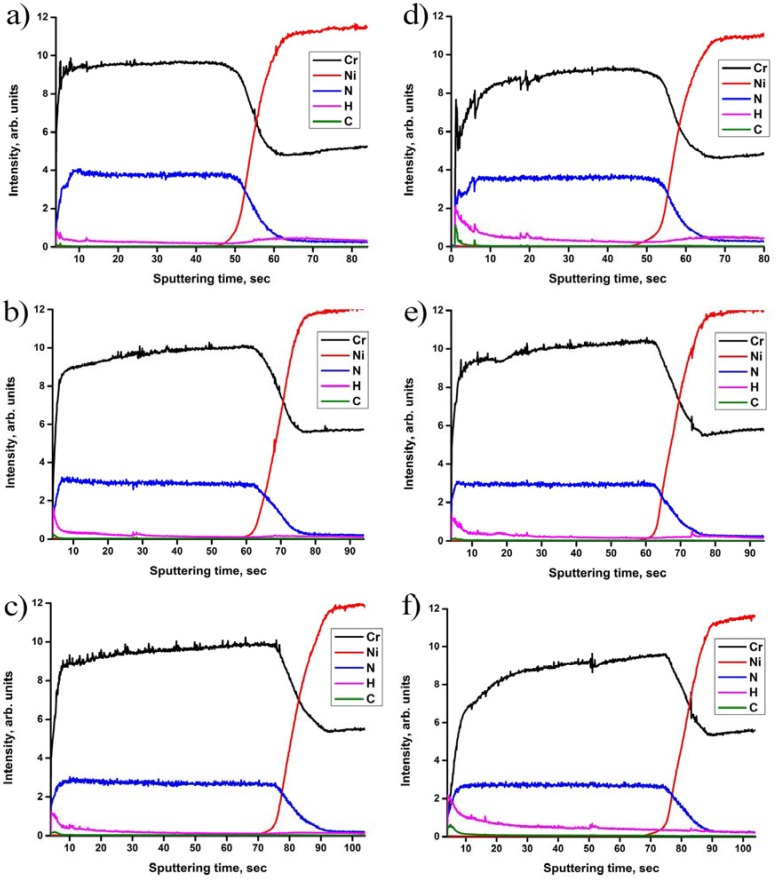
GDOES profiles of elements at (**a**) 300 mPa; (**b**) 600 mPa; (**c**) 900 mPa as-deposited; and (**d**) 300 mPa; (**e**) 600 mPa; (**f**) 900 mPa hydrogen exposed Cr_x_N coatings on the IN718 substrates.

Figure 4 shows the depth distribution of elements in the samples before and after hydrogen exposure at 600 °C and 2 atm hydrogen pressure for 2 h. A uniform distribution of the elements Cr and N is observed through the depth of the as-deposited Cr_x_N coatings.

The Cr to N ratio increases with pressure. These results are in agreement with the WDS measurements. Fluctuations of the signal intensities in the initial stage are associated with surface contaminations. The hydrogen concentration in the as-deposited coatings is at the same low level, like in the bulk IN718 alloy, which is typically only a few ppm.

After hydrogen exposure, the hydrogen concentration in the coatings does not change significantly (Figure 4d–f). However, hydrogen is trapped at the surface and decreases monotonically with increasing depth to the value of the bulk alloy. Despite low hydrogen values in the GDOES profiles, a large amount of hydrogen is absorbed by the samples during gas-phase hydrogenation (see Figure 2). The reason for such low hydrogen concentration in coatings and substrate is the hydrogen evacuation from the chamber while the samples were still hot. Thus, hydrogen was desorbed during final cooling. Comparison of the results shows that after hydrogen exposure and slow cooling, concentration and distribution of the residual hydrogen is similar for the coatings deposited at different pressures. These measurements indicate that the absorbed hydrogen does not form strong chemical bonds with the coating elements at these conditions. This result was also confirmed by XRD.

### 3.5. Mechanical Properties

**Figure 5:**
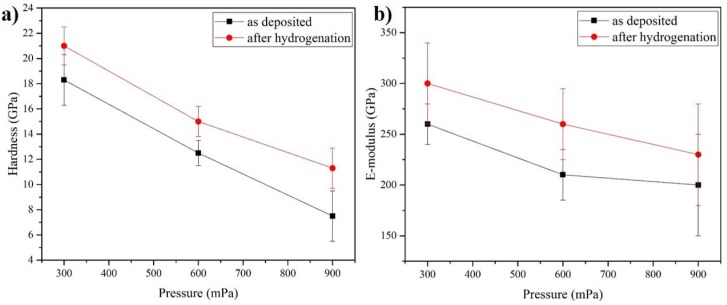
Mechanical properties (**a**) hardness; and (**b**) e-modulus of Cr_x_N coatings at various argon pressure as-deposited and after hydrogen exposure.

The mechanical properties of the coatings were characterized by measuring hardness (*H*) and elastic modulus (*E*). Values for hardness and elastic modulus of the as-deposited coatings at different pressures decline with increasing chamber pressure from 18.2 GPa (*H*) and 260 GPa (*E*) at 300 mPa to 7.6 GPa (*H*) and 200 GPa (*E*) at 900 mPa (Figure 5). According to literature, the Cr_2_N phase has better mechanical properties compared to CrN [18,41]. The mixed Cr_2_N + CrN structure deposited at 300 mPa exhibits higher hardness than Cr_2_N deposited at 900 mPa. This is likely related to the change in structure and texture within the coatings. The coating deposited at a low pressure exhibits a denser columnar structure than the one deposited at a high pressure. This leads to a lower hardness of the coating due to incompactness of the columnar structure at a higher pressure [39,42]. Furthermore, residual stresses strongly affects the mechanical properties of coatings [43]. The hardness measured for coating deposited at 900 mPa is lower than the one reported in literature for Cr_x_N coatings [18]. The Cr_x_N films after hydrogen exposure exhibit higher hardness as well as elastic-modulus and reach a maximum of 21 GPa (*H*) and 300 GPa (*E*) for coating deposited at 300 mPa. This result could be explained by invasion of hydrogen atoms in lattice vacancies inducing lattice distortion in crystal lattice and grain boundaries. Such hardening was observed for AlN coatings after hydrogen permeation, also [44]. Furthermore, hydrogen-induced defects could appear after hydrogen interaction with the coating causing the increase in hardness of the coating. Intensive study of the defect structure of the coatings is required to determine the mechanisms of improving hardness.

### 3.6. Tribology

**Figure 6:**
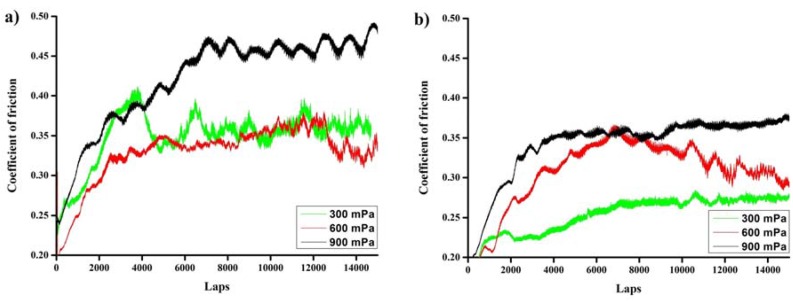
Evolution of friction coefficient for (**a**) as-deposited and (**b**) hydrogen exposed Cr_x_N coatings as a function of argon pressure.

The results of tribological investigations of the Cr_x_N-coated samples before and after hydrogenation are presented in Figure 6. The evolution of the friction coefficient with increasing distance is typical for Cr–N coatings, which is described by Rapoport et al. [45]. As reported previously [39], the friction coefficient for CrN is lower than for Cr_2_N. Moreover, according to J. Lin et al. [46] the lowest coefficient was observed in the films containing a mixture of hcp-Cr_2_N and c-CrN phases. In our case, the lowest values of CoF 0.35 and 0.34 were achieved for Cr_x_N deposited at 300 and 600 mPa, exhibiting a mixture of phases. The friction coefficient increases with pressure up to 0.45 for the coating deposited at 900 mPa. This is likely attributed to the low hardness and highly porous structure of the coating. The sharp changes in the CoF curves for the deposited coatings at the initial stage are characterized by the ploughing friction arising from the micro asperities of the surface. The small powder-like particles are generated from wear debris after the initial stage of sliding. The entrapped hard wear particles can cause ploughing friction and the occurrence of negligible interlocking effects. With increase in quantity and size of the wear particles, due to third-body hard particles the friction mechanism is ploughing [47,48]. Thus, fluctuations and rapid increase in friction coefficient occur at the end of the test. After hydrogenation, the Cr_x_N coatings show (by 20%–30%) a lower friction coefficient for the samples deposited at 300 and 900 mPa, except for the coating deposited at 600 mPa, which average CoF slightly decreased due to another wear character. The best CoF value was 0.25 for the hydrogen exposed Cr_x_N coating deposited at 300 mPa. The decrease in the CoF after hydrogenation could be attributed to the increase in hardness and Young's modulus of the coatings. It was shown that the coefficient of friction and wear resistance of coatings depends on the hardness. In particular, there was a decrease in wear resistance of the CrN coatings with a decrease in hardness [49,50]. The coefficient of friction increases when decreasing the hardness of the coating due to an increase of the area of intimate contact between the surface and the counterpart. Until now, the exact reason for the changes in CoF for the deposited coating is not clearly understood and requires further investigations.

## 4. Conclusions

Cr_x_N coatings were deposited on the IN718 substrates at working pressures between 300 and 900 mPa using DC reactive magnetron sputtering. The deposition rate increases with increasing pressure, whereas the Cr/N ratio changes from 1.4 to 1.9. XRD analysis shows the change from mixed hcp-Cr_2_N and fcc-CrN structure to approximately stoichiometric Cr_2_N with increasing working pressure. Hardness and elastic modulus of the as-deposited coatings decrease with increasing pressure. The hydrogen absorption at 600 °C for all the coatings is similar and the lowest hydrogen absorption rate was observed for the Cr_x_N coating deposited at 300 mPa. After hydrogen interaction, the phase composition of the coatings remains stable. It could be concluded that the presence of c-CrN in a mixed structure is preferable for hydrogen permeation resistant coatings, due to the higher packing fraction of cubic structure in comparison to the hexagonal one. After hydrogen exposure, the Cr_x_N films exhibit higher hardness as well as elastic-modulus and reach the highest value of 21 GPa (*H*) and 300 GPa (*E*) for the coating deposited at 300 mPa. After hydrogenation, Cr_x_N coatings show a lower (by 20%–30%) friction coefficient that favorably affects wear resistance. The best value of CoF was 0.25 for hydrogen exposed Cr_x_N coating deposited at 300 mPa.

## Figures and Tables

**Table 1:** WDS measurements obtained for the Cr_x_N coatings at various pressures.

Element (at. %)	Pressure, mPa		
	300	600	900
Cr	59	62	66
N	41	38	34
